# Patterns of Caregiver Communicative Behaviors Among Low-Income Chinese Immigrant Mothers of Children with Autism—An Exploratory Study

**DOI:** 10.3390/bs15121693

**Published:** 2025-12-06

**Authors:** Yue Xu, Xian Kapetanakos, Madeleine Meehan, Jocelyn Tam, Sandra Beatriz Vanegas

**Affiliations:** 1 University of Illinois College of Medicine, Rockford, IL 61107, USAjtam8@uic.edu (J.T.); 2 School of Social Work, Adelphi University, Garden City, NY 11530, USA; xkapetanakos@adelphi.edu; 3 Steve Hicks School of Social Work, University of Texas Austin, Austin, TX 78712, USA; sandra.vanegas@austin.utexas.edu

**Keywords:** caregiver-child interaction, autism, joint engagement

## Abstract

Caregiver communicative behaviors are critical in supporting social and language development in children with autism, yet little is known about how these behaviors manifest among Chinese immigrant families who face unique cultural and socioeconomic challenges. This study examined the communicative strategies of 11 Chinese immigrant caregivers of preschool-aged children with autism in the US during structured caregiver–child interactions. Caregiver behaviors were coded across directive and non-directive categories, including supportive directives, directives, labeling, praise, imitation, and expansion, and joint engagement quality was rated using the Joint Engagement Rating Inventory (JERI). Results showed that supportive directives and directives were the most frequent behaviors, reflecting cultural values of parental guidance and educational scaffolding, whereas non-directive strategies such as imitation and expansion were less common and more often observed among higher-income and more acculturated families. Caregiver self-efficacy in using evidence-based strategies was positively associated with greater use of non-directive communicative strategies and higher joint engagement scores. Results suggest that providers should recognize and build on culturally grounded strengths, such as the educator role and calm authority, while introducing complementary strategies that enhance joint engagement. Culturally and linguistically responsive support is especially needed to ensure equitable access for families with limited English proficiency or lower income. Although limited by a small sample size, this exploratory study provides preliminary insight into culturally influenced caregiver–child communication patterns and offers directions for larger, more rigorous research.

## 1. Introduction

Early identification and intervention are crucial to language and behavior development in children with autism. One of the primary characteristics of autism is a delay in the development of social communication and interaction (Golson et al., 2022). Studies have shown that non-speaking children with autism are far more likely to develop verbal language skills if they begin early intervention services when they are younger than five years old, compared with children who begin past five years old (Landa, 2018). Early intervention for children with autism has been demonstrated by numerous studies to prevent challenging behaviors such as aggression, tantrums, and self-injury (Koegel et al., 2014). Family plays a crucial role in early intervention with children diagnosed with autism, including but not limited to accepting the autism diagnosis, understanding the child’s behaviors, facilitating interventions, and enhancing communication with the child (Bernier et al., 2010; Gardiner & French, 2011). 

Caregiver and child interactions have a large impact on the developmental outcomes of children with autism; many studies have shown a reduction in challenging behaviors with increased quality and quantity of caregiver interaction (Gao et al., 2024; Kasari et al., 2014; Landa, 2018; McDuffie & Yoder, 2010; Siller & Sigman, 2008). Caregiver-to-child communication is especially crucial for language development in children with communication or speech delays (Levickis et al., 2014). Western mothers (e.g., United States) have been shown to interact differently with their children with autism when compared to interactions with neurotypical children (Kasari et al., 2014). Here, “Western” refers to sociocultural contexts shaped by Euro-American norms that prioritize individualism, autonomy, and child-led interaction. It is also known that in low-resource households, the quality and quantity of parent–child interaction are factors that significantly impact the language development in children (Hirsh-Pasek et al., 2015; Roberts & Kaiser, 2011). Due to the profound impact that the quality and responsiveness of caregiver communication has on children with autism, individualized and parent-centered interventions should continue to be explored as low-cost and high-efficacy models (Roberts & Kaiser, 2011).

It is crucial to understand how caregivers communicate with their children in immigrant communities, as 22 percent of the United States population speaks a language other than English at home (*Language spoken at home*, n.d.). However, caregiver-to-child communication has not been studied in the intersectional community of low-resourced immigrant households of children with autism. In recent years, the number of racially and ethnically diverse children who are identified and diagnosed early has grown (Shaw et al., 2025), and the literature on adapting interventions for various cultural groups has expanded (Magaña et al., 2021; Lindly et al., 2025; Xu et al., 2022). However, little is known about how parents interact with their children with autism (Lee et al., 2025; Palmer & Riley, 2025). Immigrant families are underserved and underrepresented in research and interventions for autism (Lee et al., 2025) and can feel extra stress in obtaining services due to communication barriers, disrespect of values, and lack of understanding (Son et al., 2017; Xu et al., 2022). Therefore, addressing this gap in knowledge about diverse caregiver–child interaction styles is essential for designing culturally responsive and more individualized interventions.

Culture encompasses many aspects of a family’s identity and shapes the way people within a cultural group communicate with one another verbally and nonverbally (Golson et al., 2022). While this is true for all languages, families who speak a language other than English may bring communication norms—such as different expectations for directness, deference, or nonverbal cues—that do not always align with systems that operate according to mainstream English-language communication norms (Gardiner & French, 2011). Parent involvement in learning also varies based on the family’s culture and can function very differently across sociocultural backgrounds (Grinker et al., 2013; Yamamoto et al., 2022). Interventions adapted for diverse cultures have been supported by research to improve social, emotional, and behavioral skills in children with autism (Palmer & Riley, 2025). Acceptance and sustained use of interventions are higher when the intervention is culturally responsive, as well as parent-level outcomes, such as mental health and self-confidence in implementation (Lee et al., 2025). 

Immigrant families face unique challenges in navigating services and support for their children with autism, including an unfamiliar healthcare system, limited English proficiency, and lack of health insurance or being underinsured (S. C. Lin et al., 2012). Among these families, Chinese immigrants represent the largest percentage of the Asian American immigrant population and the third largest immigrant group in the United States. Notably, most children of Chinese ethnicity grow up in an immigrant household in the US (Yamamoto et al., 2022). Furthermore, Chinese immigrants have comparable rates of poverty to other immigrants and higher rates than people born in the United States (*Chinese immigrants in the United States*, 2025). Adding another layer to this context, data from the CDC’s Autism and Developmental Disabilities Monitoring Network states the prevalence of children diagnosed with autism in the United States to be the highest amongst Asian and Pacific Islanders (Shaw et al., 2025). It is important to understand how Chinese immigrants communicate with their children in order to better tailor autism interventions and parent coaching. 

There is limited research on caregiver–child communication behaviors among Chinese immigrants raising young children diagnosed with autism. However, more general studies on communication between Chinese parents and neurotypical children show large differences when compared to their European American counterparts (Xia, 2024; Q. Zhang & Wills, 2016). For example, Chinese parents emphasize education and are typically very actively involved in their child’s education, providing more structured and formal education in the home (Xia, 2024). In addition, those with higher socioeconomic status engage more frequently in language and cognitive activities, while placing less emphasis on social–emotional skills than on learning-related skills (Xia, 2024). Furthermore, a study comparing parent–child communication in the United States and China found that Chinese parents were perceived as expressing less overt affection toward their children (Q. Zhang & Wills, 2016); however, this likely reflects cultural differences in how love is demonstrated, as in Chinese culture, affection is often conveyed through subtle actions, care, sacrifice, and investment in the child’s well-being and education (S. T. Russell et al., 2010). While these subtle forms of affection are deeply valued within the culture, they may be more difficult for some children with autism to interpret, potentially influencing the dynamics of caregiver–child interactions. Taken together, these findings highlight the need to examine communication patterns within the Chinese immigrant community as they relate specifically to children with autism, particularly in the context of caregiver–child interactions. Therefore, the goal of the current study is to characterize caregiver-child interactions among low-resource Chinese immigrant families of children with autism in the US. We analyzed observational data collected at baseline as part of a broader pilot intervention study from 11 caregiver-child dyads. Only pre-intervention data are included in the present analysis. Given the limited research in this population and the small sample available, this study is intended as preliminary and exploratory, aimed at generating hypotheses and identifying culturally grounded patterns.

## 2. Methods

### 2.1. Participants

Eleven Mandarin-Speaking Chinese mothers and young children under the age of 5 with autism from Chicago and New York metropolitan area were recruited as part of a pilot parent psychoeducational intervention study with Chinese immigrant caregivers (Xu et al., 2025). To meet the inclusion criteria, caregivers needed to speak Mandarin or Cantonese fluently. Parents reported their children’s diagnoses, and the CARS-2 was used to confirm symptom presentation and level of support needs. The study was open to all primary caregivers, including mothers and fathers. However, despite outreach to both parents, all individuals who enrolled and consented to participate were mothers.

Table 1 summarizes the demographic characteristics of the caregivers and their children on the autism spectrum. All caregivers were mothers. The children had an average age of 3.6 years, and most (9 out of 11) were male. All children were born in the United States and were, on average, diagnosed at 26 months of age. Seven of the eleven children were identified as having high support needs.

The mothers were, on average, 33.5 years old and had lived in the United States for approximately 12 years. Most (6 out of 11) reported speaking English “not well.” Educational attainment varied: four had a high school education or less, four had some college or a bachelor’s degree, and three held graduate degrees. Most families (6 out of 11) had a household income below $35,000, based on the 138% federal poverty line threshold for a family of four. Six mothers were employed, and ten were married or living with a partner.

### 2.2. Data Collection Procedures

We collected Zoom recordings of parent–child dyads for 15 min during the baseline data collection of the pilot intervention study. Parents were given six options to choose from for each five minutes of the 15 min: playing with toys, playing with mother, mealtime (or snack), sharing a book, caregiving (e.g. washing hands, brushing teeth), and household chores (Dow et al., 2020). Parents selected three of the activities to participate in during the recording facilitated by trained research staff. All recordings were collected in Mandarin Chinese. 

### 2.3. Measures

*Demographic Characteristics*. Several caregiver and child demographic characteristics were included in the analysis, such as caregiver age, educational attainment, English fluency, length of residence in the United States, and the child’s age at diagnosis. Educational attainment included various levels of education ranging from “less than high school” = 1 to “graduate degree” = 6. English fluency levels include, “speaking not well”=1, “Speaking well” = 2, to “Speaking very well” = 3. 

*Level of Support Needs*. The level of support needs for children with autism was measured using the Childhood Autism Rating Scale Standard Version 2nd edition (Schopler et al., 2010) (CARS2-ST). CARS is a validated instrument used to assess the severity of autism-related symptoms. It consists of 15 items that evaluate various aspects of behavior, including frequency, intensity, duration, and atypicality of social communication challenges and other behaviors. Each item is rated on a four-point scale, with detailed descriptors guiding administrators in assigning scores. A Chinese version of CARS has been translated and psychometrically validated (Lu et al., 2004). Consistent with thresholds used in prior research involving comparable assessments (S. H. Kim et al., 2019), an interrater agreement benchmark of 80% was established for initial reliability. To achieve this, two trained graduate researchers jointly coded 25% of the recordings (seven parent–child dyads). The remaining videos were independently coded by either of the two raters.

*Caregiver Self-Efficacy in Using Evidence-Based Strategies*. Self-efficacy in applying evidence-based strategies (EBS) was measured using a 10-item questionnaire developed by the Parents Taking Action (PTA) research team (Magaña et al., 2017). Participants responded on a four-point Likert scale ranging from (1) “strongly disagree” to (4) “strongly agree.” Total scores were calculated by summing item responses, with higher scores indicating greater self-efficacy in using the strategies. The questionnaire was translated and back-translated following Brislin’s method (Brislin, 1970).

*Caregiver Communicative Behavior*. Caregiver communicative behaviors were coded using a micro-analytic coding scheme, which involves logging each individual communicative act as it occurs during the interaction. This moment-by-moment approach allows for a fine-grained analysis of how caregivers initiate and respond to their child’s behaviors using predefined behavior categories (Bakeman & Gottman, n.d.). Coders categorized each caregiver utterance or nonverbal act as one of several communicative behavior types: directive, praise, expansion, imitation, responsive question, supportive directive, label, or missed opportunity. Definitions followed those described in Conway et al. (Conway et al., 2018), which draws from prior validated coding systems (Akhtar et al., 1991; Levickis et al., 2014). Missed opportunity is defined as the caregiver not responding in any way to a child’s utterance after 3 s.

Two trained coders independently reviewed the full 15 min free-play videos and recorded all caregiver communicative acts. Frequencies were converted to rates per minute for each behavior type. To establish interrater reliability, 20% of the videos were double-coded, and coders achieved intraclass correlation coefficients above 0.80 before proceeding independently.

*Joint Engagement Rating Inventory*. After coding communicative behaviors, trained research assistants rated the quality of caregiver–child social interaction using selected items from the Joint Engagement Rating Inventor (JERI) (Adamson et al., 2020). The following seven categories were rated on a 7-point Likert scale: Fluency and Connectedness, Caregiver Scaffolding, Caregiver Affect, Caregiver’s Following on Child’s Focus, Caregiver’s Calm Authority, Caregiver’s Language Facilitation, and Caregiver’s Communicative Temptation. Each 15 min free-play session was divided into three 5 min segments. Two independent coders rated each segment, and their scores were averaged for each item per segment. The segment scores were then averaged across the three intervals to produce a final overall score for each category per caregiver–child dyad.

All coders were trained using standardized procedures outlined in the JERI manual. Interrater reliability was assessed on 20% of sessions, with coders achieving an intraclass correlation coefficient (ICC) of ≥0.87 before proceeding with independent coding.

### 2.4. Analysis

The research assistants used ELAN annotation tool to code the caregiver communicative behaviors (directives, praise, expansion, imitation, responsive questions, supportive directives, label, missed opportunity) adapted from Conway’s study (Akhtar et al., 1991). After the coding of the video, the calculations were performed to show the total number of each behavior and rate per minute for each behavior. 

After coding communicative behaviors, the research assistant rated the social interaction on a seven-point scale based on the Joint Engagement Rating Inventory (Adamson et al., 2020). The categories were fluency and connectedness, caregiver scaffolding, caregiver affect, caregiver’s following in on child’s focus, caregiver’s calm authority, caregiver’s language facilitation, and caregiver’s communicative temptation. The summary scores for each 5 min segment were calculated and averaged out for the overall score.

Given the small sample size, we operated a non-parametric correlation analysis Kendall’s Tau-B using SPSS 27 to evaluate the association of caregiver and child characteristics with (1) caregiver communication behaviors, and (2) joint engagement quality. 

## 3. Results

### 3.1. Caregiver Communication Patterns and Engagement Quality

**Figure 1:**
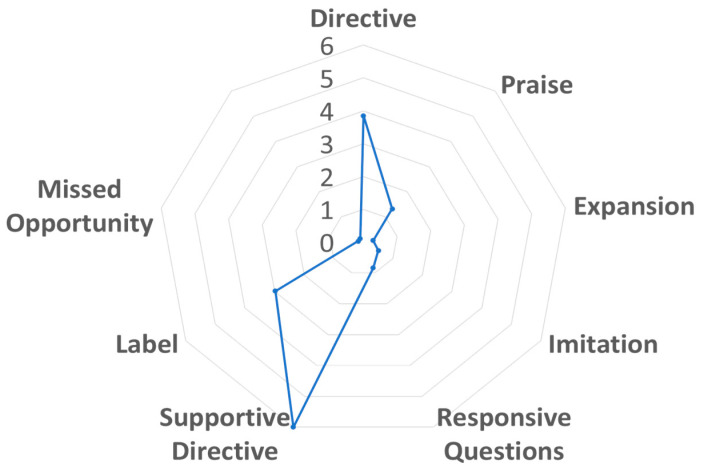
Average Frequency of Caregiver Communicative Behavior.

Given the preliminary and exploratory nature of the study, the findings below highlight patterns rather than generalizable estimates. Table 2 displays the summary statistics for caregiver communicative behaviors and engagement quality during parent–child interactions. The most frequently observed communicative behaviors were supportive directives (M = 6.0 per minute, SD = 3.1) and directives (M = 3.9, SD = 2.9), followed by labeling (M = 3.0, SD = 2.7) and praise (M = 1.3, SD = 0.9). Less frequent behaviors included responsive questioning (M = 0.8, SD = 0.7), imitation (M = 0.5, SD = 0.4), expansion (M = 0.3, SD = 0.4), and missed opportunities (M = 0.2, SD = 0.2). Figure 1 visually depicts the different aspects of caregiver communicative behavior observed during caregiver–child play interactions. The chart highlights the relative strengths and areas that need support among participating caregivers.

In terms of joint engagement quality, caregivers scored highest in language facilitation (M = 4.4, SD = 1.5), scaffolding (M = 4.3, SD = 1.3), and calm authority (M = 4.3, SD = 1.1). Following the child’s focus (M = 4.0, SD = 1.1), affect (M = 3.9, SD = 1.6), and communication temptation (M = 3.7, SD = 1.5) were rated moderately, while fluency and connectedness had the lowest mean rating (M = 3.6, SD = 1.2) among the engagement dimensions.

### 3.2. Correlations Between Caregiver and Child Characteristics and Communicative Behaviors and Joint Engagement

Table 3 presents Kendall’s Tau-b correlations between caregiver and child characteristics and caregivers’ observed communicative behaviors. Children’s level of support needs (CARS) was positively correlated with directive communication (τ = 0.65). Caregiver self-efficacy was positively correlated with praise (τ = 0.46), expansion (τ = 0.38), and responsive questions (τ = 0.34).

English fluency was positively correlated with expansion (τ = 0.26) and imitation (τ = 0.20), and negatively correlated with directive (τ = −0.60) and supportive directive behaviors (τ = −0.57). Higher caregiver education and self-efficacy were negatively correlated with missed opportunities during interactions. Income was positively correlated with responsive questions (τ = 0.54) and negatively with directive behaviors (τ = −0.56).

Table 4 shows Kendall’s Tau-b correlations between caregiver and child characteristics and observed joint engagement ratings. Caregiver self-efficacy was positively correlated with all joint engagement dimensions, including scaffolding (τ = 0.88), affect (τ = 0.69), following child’s focus (τ = 0.66), calm authority (τ = 0.75), language facilitation (τ = 0.85), and communication temptation (τ = 0.76). No significant associations were found between CARS scores and joint engagement variables. In addition, income is positively correlated with following child’s focus (τ = 0.56). Age of diagnosis was negatively correlated with affect (τ = 0.52) and language facilitation (τ = −0.53)

## 4. Discussion

The current study sought to examine the general characteristics of caregiver communicative behaviors and the quality of joint engagement among low-income Chinese immigrant mothers with young children diagnosed with autism. Additionally, the study aimed to investigate factors associated with caregiver communicative behaviors, including family income, caregiver characteristics (e.g., education level, acculturation), and child-specific factors (e.g., language abilities, level of support needs). As a preliminary exploratory study with a small sample, our findings should be interpreted as early insights that point to culturally relevant directions for future research. By exploring these associations, the study seeks to deepen the understanding of how sociocultural and contextual variables influence mother-child interactions in this underserved population. Furthermore, the knowledge gained from this research aims to inform the identification of strengths and culturally responsive targets for early intervention efforts. Although cultural patterns are referenced to contextualize findings, Chinese immigrant families are highly heterogeneous, and the associations observed in this study reflect considerable within-group variability.

The findings of this preliminary study indicate a significant association between the level of support needs and the frequency of parental use of directive communication strategies during interactions. Consistent with existing literature, parents of children with more pronounced autism-related challenges may be more likely to adopt a more directive communication style as a means of facilitating engagement and guiding their child’s participation (Loncarevic et al., 2024). Importantly, this relationship should not be misconstrued as indicating that parental directiveness contributes to the emergence of autistic behaviors. Rather, it is likely that the child’s social-communication difficulties, such as limited attention span, reduced reciprocal engagement, and challenges in initiating interactions, prompt parents to employ more directive strategies to achieve desired interaction outcomes (Edmunds et al., 2019; McCauley & Solomon, 2022). In this context, parental directiveness may function as an adaptive and responsive approach, designed to scaffold the child’s participation and support interaction flow (McCauley & Solomon, 2022). Nevertheless, while directive communication may serve as a practical tool to manage immediate interactional demands, excessive reliance on such strategies could inadvertently constrain opportunities for reciprocal exchanges that are essential for fostering social-communication development (Crowell et al., 2019; Fernández Cerero et al., 2024). Therefore, the severity of a child’s autism symptoms may shape parental communicative patterns, leading to a greater emphasis on directive interactions as parents navigate the complexities of supporting their child’s engagement and regulation needs (Suvarna et al., 2024). Finally, the use of brief, structured 15 min caregiver–child observations conducted over Zoom may have introduced bias into the behaviors captured in this study. Structured tasks inherently invite more parent guidance and instructional scaffolding, which may amplify the appearance of directive behaviors. Remote observation may also heighten parents’ tendency to ‘perform’ expected teaching behaviors, particularly among Chinese immigrant caregivers who culturally emphasize the educator role, although there is considerable diversity in how Chinese families interpret and enact this role. As a result, the relative frequency of directive versus non-directive strategies in our dataset may not fully represent families’ everyday, naturalistic communication. 

The income level of Chinese immigrant mothers was found to be positively associated with their use of responsive questioning and their ability to follow their child’s focus during parent–child interactions (X. Lin & Yang, 2024). One possible explanation for this association is that higher-income parents often have greater access to educational resources, parenting information, and early intervention services, which may increase their awareness of developmentally supportive communication strategies (Hill, 2006). As a result, mothers with higher socioeconomic status may be more inclined to adopt a responsive, child-centered approach, emphasizing techniques such as following the child’s lead and engaging in play-based interactions (X. Lin & Yang, 2024). These may have greater access to information about the importance of fostering reciprocal exchanges and promoting their child’s autonomy during interactions, which are key components of responsive questioning and attention-following behaviors (Sengonul, 2022).

In addition to higher income being associated with increased use of responsive questioning and child-focused interactions, caregiver’s English proficiency was also found to have a significant association with communicative behaviors (Ortega & Ludwig, 2023). Specifically, caregivers with higher levels of English proficiency were more likely to engage in expansion and imitation strategies, while exhibiting lower frequencies of directive and supportive directive communication. This pattern may reflect differences in acculturation levels among immigrant parents. Mothers with stronger English skills are likely to have resided in the host country for a longer period, providing them with greater exposure to child-centered interaction styles that emphasize following the child’s lead, encouraging autonomy, and utilizing responsive communication strategies (Glick et al., 2012). These parents may be more familiar with Western parenting approaches that prioritize mutual engagement and shared meaning-making in interactions (Bornstein, 2012). In contrast, parents with limited English proficiency, who may be more recent immigrants, tend to rely more on directive and supportive directive behaviors (Schofield et al., 2012). This inclination may stem from culturally rooted values in traditional Chinese parenting, which emphasize adult-led instruction, obedience, and structured guidance through didactic communication (Huang et al., 2017; X. Zhang et al., 2024). For these parents, a more directive approach aligns with their cultural expectations of effective teaching and caregiving, even within the context of a new cultural environment (Cheah et al., 2015; Chen & Huang, 2025).

Maternal self-efficacy in implementing evidence-based strategies (EBS) was found to be significantly and positively correlated with all measures of joint engagement (Keen et al., 2010). When mothers hold strong beliefs in their ability to effectively support their child using EBS, they are more likely to engage in interactions that are synchronized, reciprocal, and sustained—key components of joint engagement that are critical for promoting developmental outcomes in children with autism (Keen et al., 2010). High self-efficacy empowers mothers to confidently apply strategies that foster shared attention, mutual responsiveness, and extended interactive episodes, which are foundational for supporting their child’s social communication and learning (Kurzrok et al., 2021). This finding highlights the pivotal role of caregiver confidence in facilitating meaningful parent–child interactions and underscores the importance of building parents’ competence and belief in their ability to implement intervention strategies effectively as a core component of early intervention programs. It is also possible that higher self-efficacy reflects greater access to services, coaching, or educational resources rather than self-efficacy alone driving interaction quality.

Given the influence of acculturation on parental communicative behaviors, it is essential for parent-mediated intervention programs to consider the cultural backgrounds and acculturation levels of immigrant families when designing and delivering interventions (Lopez, 2024; Shorey et al., 2020). Parents with higher levels of acculturation, including stronger English proficiency and longer residency in the host country, may be more receptive to child-centered strategies that emphasize following the child’s lead, fostering autonomy, and promoting reciprocal interactions. Conversely, parents who are less acculturated, particularly recent immigrants with limited English proficiency, may feel more aligned with traditional, adult-led approaches that prioritize direct instruction and structured guidance (S. Y. Kim et al., 2013). Interventions that overlook these cultural preferences may face challenges in parent engagement and implementation fidelity. Therefore, culturally responsive interventions should incorporate flexible strategies that respect and build upon parents’ existing beliefs and practices while gradually introducing responsive, child-centered techniques. Providing culturally sensitive explanations of the rationale behind these strategies, offering bilingual resources, and using culturally congruent examples can facilitate better understanding and acceptance among parents (Yang & Crehan, 2023). Furthermore, interventionists should adopt a collaborative coaching approach that validates parents’ cultural values, while supporting them in integrating new interaction styles that enhance their child’s social-communication development. Recognizing acculturation as a dynamic and individualized process is crucial for tailoring interventions to meet the diverse needs of immigrant families effectively (Kosic, 2025).

The strong association between caregiver self-efficacy in using evidence-based strategies (EBS) and higher quality joint engagement underscores the critical importance of fostering parental confidence as a foundational element in intervention design (Keen et al., 2010). Intervention programs that prioritize enhancing parents’ belief in their ability to support their child’s development can create a positive feedback loop, where increased self-efficacy leads to more effective caregiver-child interactions, thereby reinforcing caregivers’ confidence and commitment to the intervention process (Fante et al., 2024). Parent coaching models should thus incorporate structured opportunities for parents to experience success in applying EBS within everyday routines, providing immediate, specific feedback that validates their efforts and builds their competence. 

Furthermore, interventions should adopt a strengths-based approach, emphasize parents’ existing skills while scaffold new strategies in a supportive, collaborative manner. By empowering parents through guided practice (Lorio et al., 2021), modeling, and reflective coaching conversations (Artis et al., 2022), practitioners can enhance parental agency, promote sustained engagement in intervention activities, and ultimately improve developmental outcomes for children with autism. Recognizing self-efficacy as a modifiable factor is key to designing culturally sensitive, family-centered interventions that equip parents with the confidence and skills needed to foster meaningful and developmentally supportive interactions with their children (K. M. Russell & Ingersoll, 2021).

### 4.1. Implication for Policy and Practice

Although these associations are correlational and should not be interpreted causally, they offer preliminary insights that may help guide practice. Findings from this study highlight several important considerations for supporting Chinese immigrant families of young children on the autism spectrum, particularly in promoting joint engagement through caregiver–child interactions. First, for families of children with more severe autism symptoms—who may struggle with sustained engagement—providers should introduce strategies beyond directiveness. Approaches such as imitation, language expansion, following the child’s lead, and communication temptations may offer alternative pathways to fostering shared attention and reciprocity. Second, professionals working with Chinese caregivers should recognize and build on culturally grounded strengths. Many caregivers in this study demonstrated an “educator” role, characterized by scaffolding, supportive directives, and a calm, structured presence. These behaviors can serve as a strong foundation for introducing additional evidence-based strategies in ways that feel culturally congruent and respectful.

However, our findings also revealed disparities: caregivers with higher income and greater acculturation were more likely to use responsive strategies like imitation and expansion. This suggests a critical equity gap. Policies and service systems should prioritize accessible, linguistically appropriate, and culturally tailored supports for low-income Chinese immigrant families with limited English proficiency. Strengthening interpreter services, expanding bilingual service delivery models, and supporting the development of intervention materials in families’ preferred languages are key steps toward reducing language-related barriers. Additionally, improving equity in access to early intervention, such as reducing waitlists, increasing availability of culturally and linguistically matched providers, and streamlining the navigation process, can ensure that families with fewer resources have the opportunity to benefit from strategies that promote meaningful engagement and developmental growth. It is important to note that Chinese immigrant families vary widely in parenting beliefs and resources, and income may intersect with many contextual factors that differ across households.

### 4.2. Limitations

This study has several limitations that should be acknowledged when interpreting the findings. First, the sample size was small and limited to Chinese immigrant mothers in urban settings, which may restrict the generalizability of the results to other cultural groups or immigrant communities. Second, the observational data were based on brief parent–child interactions within structured settings, which may not fully capture the range and variability of communication behaviors used in everyday routines. The structured nature of the observation may lead to more directive interactions between the caregiver and child. Because only mothers chose to participate in this study, the perspectives of fathers and other caregivers are not represented, which may limit the diversity of caregiving experiences captured. Additionally, the cross-sectional nature of the study prevents us from drawing causal conclusions about the relationship between caregiver characteristics and specific communication strategies. 

### 4.3. Future Directions

Despite the small sample size, there may be innovative ways to expand this line of research in a more rigorous evaluation. One promising direction is the use of visual artificial intelligence (AI) to automate the coding of caregiver–child communication behaviors (Zheng et al., 2024), a process that is traditionally time-consuming and resource-intensive. Automating behavioral coding could enable the inclusion of larger and more diverse samples, improve reliability, and facilitate real-time feedback for intervention studies. Future research could also adopt longitudinal and cross-cultural designs to examine how caregiver communication styles and self-efficacy evolve over time and across different sociocultural contexts. Additionally, rigorous intervention trials could evaluate how culturally adapted coaching approaches enhance caregiver self-efficacy and responsive communication, ultimately promoting better developmental outcomes for children with autism.

## 5. Conclusions

This study offers detailed insight into the communication behaviors of Chinese immigrant caregivers of young children with autism and highlights both cultural strengths and areas for support. While directive strategies were commonly observed, many caregivers also demonstrated a strong educator role characterized by scaffolding and a calm, supportive presence. However, more responsive strategies such as imitation and expansion were less frequently used, particularly among lower-income and less acculturated families. 

A key contribution of this study is the strong association between caregiver self-efficacy in using evidence-based communication strategies and higher ratings across nearly all joint engagement dimensions. These results suggest that caregivers who feel more confident in applying responsive, developmentally supportive strategies tend to create richer opportunities for shared attention, reciprocal interaction, and sustained engagement—core processes linked to children’s social communication and language growth. Finally, although cultural factors offer useful context, it is essential to recognize the considerable within-group diversity among Chinese immigrant families; the patterns observed in this study represent only a subset of experiences and should not be generalized across all families. Further studies with larger and more diverse samples will be essential to verify and extend the preliminary findings from this exploratory study. 

Taken together, these findings underscore the need for culturally responsive early intervention approaches that build on caregiver strengths while building confidence in using responsive, child-led strategies. Enhancing self-efficacy—particularly among families with fewer resources or lower levels of acculturation—may be a critical mechanism for increasing the frequency and developmental impact of parent–child engagement in diverse immigrant communities. 

## Figures and Tables

**Table 1:** Demographics Characteristics of Participating Caregivers and Children.

Demographic Variables	Mean or *n* (%)	SD
Child		
Age (years)	3.6	1.4
Sex		
Male	9 (82)	
Female	2 (18)	
US Born	11 (100)	
Age of diagnosis (months)	26	5.9
Autism Level of Support Needed (CARS2)		
Mild to Moderate Support	4 (36.4)	
High Support Needs	7 (63.6)	
Caregiver		
Sex		
Male	0	
Female	11 (100)	
Age (years)	33.5	5.9
Years in US	11.7	5.9
Speaks English		
Not well	6 (54.5)	
Well	3 (27.3)	
Very well	2 (18.2)	
Education Level		
High School or Less	4 (36.4)	
Some College to Bachelor’s	4 (36.4)	
Graduate Degree	3 (27.3)	
Income		
<$35,000 ^a^	6 (54.5)	
≥$35,000	5 (45.5)	
Employed	6 (54.5)	
Married or Living Together	10 (90.9)	

^a^ $35,000 is the approximate income for a family of four under 138% federal poverty line threshold at the time of data collection in 2021.

**Table 2:** Summary Statistics of Caregiver Communicative Behaviors and Engagement Quality.

Caregiver Communicative Behaviors (Rate per Minute)	Mean	Standard Deviation
Supportive Directive	6.0	3.1
Directive	3.9	2.9
Praise	1.3	0.9
Label	3.0	2.7
Responsive Question	0.8	0.7
Imitation	0.5	0.4
Expansion	0.3	0.4
Missed Opportunity	0.2	0.2
Engagement Quality (Scale 1–7)		
Fluency and Connectedness	3.6	1.2
Language Facilitation	4.4	1.5
Scaffolding	4.3	1.3
Calm Authority	4.3	1.1
Follow Child’s Focus	4.0	1.1
Affect	3.9	1.6
Communication Temptation	3.7	1.5

Note: Caregiver communicative behaviors are coded as rates per minute. Engagement quality variables are scored on a 1–7 scale, with higher scores indicating more optimal interaction quality.

**Table 3:** Correlations between Caregiver and Child Characteristics and Caregiver Communicative Behaviors.

		Supportive Directive	Directive	Praise	Label	Responsive Question	Imitation	Expansion	Missed Opportunity
Caregiver Characteristics	Income	−0.06	−0.56 *	−0.08	0	0.54 *	−0.06	0.25	−0.48
	Education	−0.14	−0.52 *	0.12	0.16	0.28	−0.08	0.32	−0.54 *
	English Fluency	−0.64 *	−0.43	−0.37	0.14	0.07	0.53 *	0.53 *	0.07
	Years in US	−0.10	−0.17	−0.08	−0.04	0.16	0.29	−0.02	0.14
	Self-efficacy in Using EBS	0.25	−0.25	0.46	0.26	0.34	−0.08	0.38	−0.61 *
Child Characteristics	Age of Diagnosis	−0.49	−0.30	−0.42	−0.14	−0.38	0.17	−0.13	0.39
	CARS	0.20	0.65 **	0.08	−0.22	−0.26	−0.25	−0.37	0.06

Note: Educational levels include less than high school = 1, high school = 2, associate degree = 3, some college = 4, bachelor’s degree = 5, graduate degrees = 6. English fluency levels include not well, well, very well. Bolded cells are statistically significant correlations. Asterisks indicate statistical significance: * *p* < 0.05, ** *p* < 0.01.

**Table 4:** Correlations between Caregiver and Child Characteristics and Joint Engagement.

		Fluency and Connectedness	Scaffolding	Affect	Following Child’s Focus	Calm Authority	Language Facilitation	Communication Temptation
Caregiver Characteristics	Income	0.36	0.27	0.18	0.56 *	0.44	0.34	0.40
	Education	0.27	0.47	0.24	0.45	0.38	0.42	0.45
	English Fluency	0.19	−0.07	0.02	0.21	−0.05	0.07	−0.02
	Years in US	0.04	−0.12	−0.14	−0.08	−0.22	−0.22	−0.24
	Self-efficacy in Using EBS	0.56 *	0.88 **	0.69 **	0.66 **	0.75 **	0.85 **	0.76 **
Child Characteristics	Age of Diagnosis	−0.36	−0.38	−0.52 *	−0.38	−0.51	−0.53 *	−0.45
	CARS	−0.23	−0.26	−0.19	−0.29	−0.14	−0.23	−0.23

Note: Bolded cells are statistically significant correlations. Asterisks indicate statistical significance: * *p* < 0.05, ** *p* < 0.01.

## Data Availability

The data presented in this study are not readily available because the video data contains identifiable visual information.

## Abbreviations

The following abbreviations are used in this manuscript:

JERI	Joint Engagement Rating Inventory
CARS	Childhood Autism Rating Scale
